# Effects of Direction and Index of Difficulty on Aiming Movements after Stroke

**DOI:** 10.1155/2014/909182

**Published:** 2014-01-28

**Authors:** Paola Ribeiro Coqueiro, Sandra Maria Sbeghen Ferreira de Freitas, Cassandra Mendes Assunção e Silva, Sandra Regina Alouche

**Affiliations:** Master's and Doctoral Programs in Physical Therapy, Universidade Cidade de São Paulo (UNICID), Rua Cesário Galeno, 448/475 Tatuapé, 03071-000 São Paulo, SP, Brazil

## Abstract

*Background.* Brain hemispheres play different roles in the control of aiming movements that are impaired after unilateral stroke. It is not clear whether those roles are influenced by the direction and the difficulty of the task. *Objective.* To evaluate the influence of direction and index of difficulty (ID) of the task on performance of ipsilesional aiming movements after unilateral stroke. *Methods.* Ten individuals with right hemisphere stroke, ten with left hemisphere stroke, and ten age- and gender-matched controls performed the aiming movements on a digitizing tablet as fast as possible. Stroke individuals used their ipsilesional arm. The direction (ipsilateral or contralateral), size (0.8 or 1.6 cm), and distance (9 or 18 cm) of the targets, presented on a monitor, were manipulated and determined to be of different ID (3.5, 4.5, and 5.5). *Results.* Individuals with right hemisphere lesion were more sensitive to ID of the task, affecting planning and final position accuracy. Left hemisphere lesion generated slower and less smooth movements and was more influenced by target distance. Contralateral movements and higher ID increased planning demands and hindered movement execution. *Conclusion.* Right and left hemisphere damages are differentially influenced by task constraints which suggest their complementary roles in the control of aiming movements.

## 1. Introduction

Hemispheric-dependent differences in the control of aiming movements have been studied by analyzing ipsilesional motor deficits after a unilateral brain damage [1, 2]. These deficits reflect the contributions of each hemisphere to unilateral arm movements that do not seem symmetrical. Individuals with left cerebrovascular accidents show significant reduction in movement speed and increased trajectory curvature [1, 2]. In fact, it was shown that only the left hemisphere controlling the dominant arm specifies movement distance by varying the early initial acceleration phase [3], which means that this hemisphere (for right handed dominant individuals) shows a specialized influence on the control of trajectory execution. On the other hand, patients with right cerebrovascular accidents show deficits in accurate execution of a planned movement [4], generating larger final position errors [1, 2, 5].

The increased movement onset time and duration are also described after left hemisphere damage on more complex motor skills [4, 6, 7]. The complexity of the movement, however, has been investigated by manipulating different factors such as the number of sequence components [8, 9], target distance [10], and accuracy constraints [4, 10, 11]. If right and left unilateral brain lesion lead to specific limb movement deficits, it is unreasonable to consider that the control of all this variety of complex motor skills could be attributed only to the left hemisphere.

One way to deal with the complexity of the task is considering the index of difficulty (ID) of the task [12]. ID is related to the movement amplitude (*A*) and the target width (*W*). Taken together, movement time (MT) would vary as a function of both aspects according to the equation MT = *a* + *b* · log⁡_2_⁡(2*A*/*W*), where *a* and *b* are constants and log⁡_2_⁡(2*A*/*W*) is considered the ID of the task. The increase in reaction time [10] and in movement time [13] for tasks with higher ID was previously demonstrated during horizontal movements of the arm on a digitizing tablet in healthy participants, and it is expected to remain constant when the relation between amplitude and width of the target is unchanged. However, a violation of this relationship was verified during bimanual movements [14], cyclic-aiming movements [15], and whole-body movements [16]. In the last case, a scaling effect of MT was observed with the movement amplitude. In this way, it is not clear whether different movement amplitudes within the same ID or whether different ID within the same distance generate changes in the performance of the task. If right and left unilateral brain damage affect the control of aiming movements differently, it is expected that left brain damage would compromise movements with higher amplitude even with the same ID, while right brain damage would affect those movements more significantly with higher ID within the same amplitude.

Furthermore, the movement direction also affects planning and execution of the aiming movements. The anticipatory control mechanisms must make use of the biomechanical properties of the limbs and of the environment to reach the target and adjust it during the movement execution [17, 18]. The directional biases to the right and left diagonal, due to different involvement of the shoulder and elbow joints [18], produce different strategies for making aiming movements [19]. Overall, aiming movements to contralateral targets of the moving arm are slower and require longer movement times and acceleration duration than those to ipsilateral targets [20]. The combined influence of both the direction and ID after unilateral brain damage was not systematically established. The effects of the different movement amplitudes with the same ID for ipsilateral and contralateral targets of the moving arm on both planning and execution of aiming movements have not been investigated. How do the right and the left hemisphere damage influence the control of ipsilesional aiming movements according to their complexity and direction?

Therefore, in the current study, we examined how the index of difficulty of the task and the direction affect the aiming movements of stroke individuals. We were interested in investigating whether the side of the hemisphere lesion influences differentially the movement behavior depending on the task constraints. Due to the specialized role of the left hemisphere in modulating the interjoint coordination and the peak velocity according to the movement amplitude on aiming movements [21], we expected that individuals with left hemisphere damage would present deficits on the execution of the task, particularly when the movement amplitude is manipulated and for contralateral targets. Conversely, the spatial constraints of the task given by the ID and the direction of movement would affect right hemisphere damaged individuals in modulating the planning aspects of the task which would predominantly influence the movement onset and its final position accuracy mainly for those tasks with higher ID.

## 2. Methods

### 2.1. Participants

The study participants included ten individuals with a single left cerebrovascular accident (LCVA) and ten with a single right cerebrovascular accident (RCVA)—located in the anterior or medium cerebral artery territory and confirmed by computed tomography or nuclear magnetic resonance image—that occurred more than 6 months prior to the experiments. Ten age-matched, healthy volunteers also participated in the experiment. All participants were 40–70 years old and right hand dominant (prior stroke for CVA groups), as determined by the Edinburgh handedness questionnaire [22]. They were required to be able to understand and follow the instructions and presented a score higher than 18 in the Mini-Mental State Exam, recommended for low or medium schooling level [23, 24]. Individuals with hemianopsia or hemineglect syndrome, which compromised task performance, were excluded. The degree of motor impairment in the upper extremity of stroke survivals was assessed by the Fugl-Meyer Motor Scale [25, 26]. The strength of the ipsilesional hand for CVA groups and both hands for controls was evaluated by using hand-grip (Jamar; Asimow Engineering Co.) and key-pinch (Preston Pinch Gauge; B & L Engineering Co.) dynamometers. A summary of the participants' characteristics is presented in Table 1. Groups were similar in all analyzed points, except for the right pinch strength which was higher (*P* = 0.01) for the control group than for the RCVA group. All participants gave informed consent consistent with the Declaration of Helsinki and according to the procedures approved by the local ethics committee.

### 2.2. Experimental Procedures

**Figure 1:**
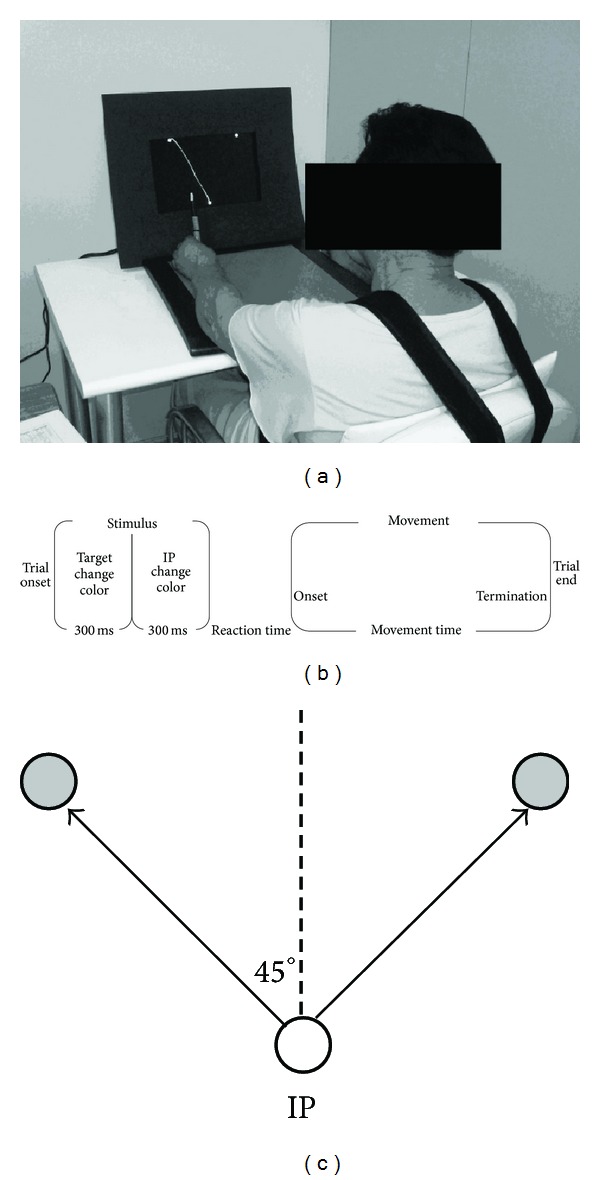
(a) Participant's position. (b) Time course of each trial. (c) Targets position (gray circles) at a distance of 18 cm and 45° to the right and left of the initial position (IP, white circle).

Participants were asked to sit on a chair in front of a table (both with adjustable height) with their trunk upright and restrained by strapping to the chair (Figure 1(a)). They maintained their upper arm aligned horizontally and the elbow flexed to approximately 90°, while holding a stylus with the hand (ipsilesional for stroke groups and right or left hand for the control group). The stylus had to be in contact with the surface of the digitizing tablet (*Wacom Intuos2*  12 × 12  *A4over D1212USB*) placed in front of the participants. Movements of the stylus led to movements of a cursor displayed on the screen of a video monitor as a 0.2 cm yellow circle. The initial position and two target locations were also displayed on the monitor screen. The trial events were composed of target stimuli (target changed from white to red and, after 300 ms, returned to white again to signalize the correct target to be reached and its position) and onset stimulus (initial position changed from white to green). At the beginning of each trial, participants were told to maintain the stylus at the initial position and to look at the screen, waiting for the stimulus at the target location. Participants were asked to start their movements from the initial position only after it became a green circle (Figure 1(b)). They were instructed to perform fast and straight movements to the target.

Discrete movements were performed toward a target placed at one of two target distances (9 and 18 cm from the initial position) and with one of two diameters (0.8 and 1.6 cm). These parameters were selected to define three indices of difficulty (ID) calculated as ID = log⁡_2_⁡(2*D*/*W*); that is, ID = 3.5, ID = 4.5, and ID = 5.5. To verify the influence of the movement amplitude over the behavior within the same ID, two combinations of target distance and dimension were used to achieve the ID = 4.5 (a target of 1.6 cm in diameter placed at a distance of 18 cm and a target of 0.8 cm in diameter placed at a distance of 9 cm). For each ID, the targets were presented at 30° to the right and left of the initial position (Figure 1(c)) and these two targets were always visible on the screen during the trials. In this way, in one block of trials, the two directions (ipsilateral and contralateral movements) could happen, randomly. Participants were encouraged to move the cursor to the center of the targets as fast as possible and this instruction was emphasized between trials.

Before the beginning of the experimental trials, the participants were allowed to familiarize themselves with the equipment as well as with the cursor trajectory. They should move the cursor to four targets placed on the screen in a different position of the analyzed conditions in order not to influence the results. This familiarization was the same for all participants. Participants performed a block of five trials for each combination of ID and amplitude, and the sequence of these blocks was randomized for each participant. A trial performed with a reaction time less than 100 ms was considered as anticipation and was rejected and repeated at the end of that specific block of trials. Stroke participants performed at least 40 trials (5 trials: 2 distances × 2 diameters × 2 directions) with ipsilesional arm, while the control group performed 80 trials in total as they moved with their right and left arms. Because only one control group was used, the first arm selected to perform the movements was alternated across participants in order to partially control the interlimb transfer effect, which is a limitation of this study, insofar that half of the control group still had practice with the other limb. An interval of 5 minutes was given between blocks, and participants could rest at any time between trials, but the participants never reported fatigue.

### 2.3. Data Analysis

The cursor trajectory was recorded at 300 Hz for posterior analysis. The trial events, data storage, and analysis were performed using a customized LabView 2009 program (*National Instruments*). Initially, the time series of the stylus trajectory (anterior-posterior and medial-lateral) were low-pass filtered at 10 Hz using a bidirectional, second-order Butterworth filter. Movement onset and termination of each trial were defined using 5% of the stylus peak velocity. The following dependent variables were assessed. Reaction time (RT) and movement time (MT) were defined, respectively, as the time elapsed from the initial position stimulus until the time when participants initiated the movement and the time from movement onset until movement termination. The peak velocity (PV) and the time between movement onset and the time of peak velocity (TPV) were also computed. The percentage of the movement time taken to reach the peak velocity was calculated. The resultant variable error was computed to assess how accurately the participants reached the targets. It was calculated by the square root of the variable error on media-lateral direction squared plus variable error on anterior-posterior direction squared. The variable error in each direction was calculated by the square root of the sum of the final position of the trajectory minus the average of the final position of all trials divided by the number of trials. The smoothness of the stylus movements was assessed by computing the number of times the stylus acceleration profile changed sign, that is, the number of zero crossing or movement units (MU). The average across trials was computed for each dependent variable and used for statistical analysis. The descriptive data are reported as means and standard error.

The normality and homogeneity of variance of data were attested by Shapiro-Wilk and Levene's Test, respectively. Repeated measure analyses of variance (RM-ANOVA) were performed to test the effects of the groups, ID (four levels) and direction (ipsilateral or contralateral targets). To test sphericity condition, Mauchly's test was used. Additionally, the *P* values were adjusted for possible deviations using Greenhouse-Geisser corrections. Separate analyses were made to include two groups at the time (i.e., RC versus RCVA; LC versus LCVA; and RCVA versus LCVA). In this way, the effect of lesion can be determined by the first two analyses and the laterality effect in the last one. SPSS version 16.0 software (SPSS, Inc., Chicago, IL) was used for analyses. When necessary, *post hoc* tests with Bonferroni adjustments were run. The level of significance was set at *P* < 0.05.

## 3. Results

**Figure 2:**
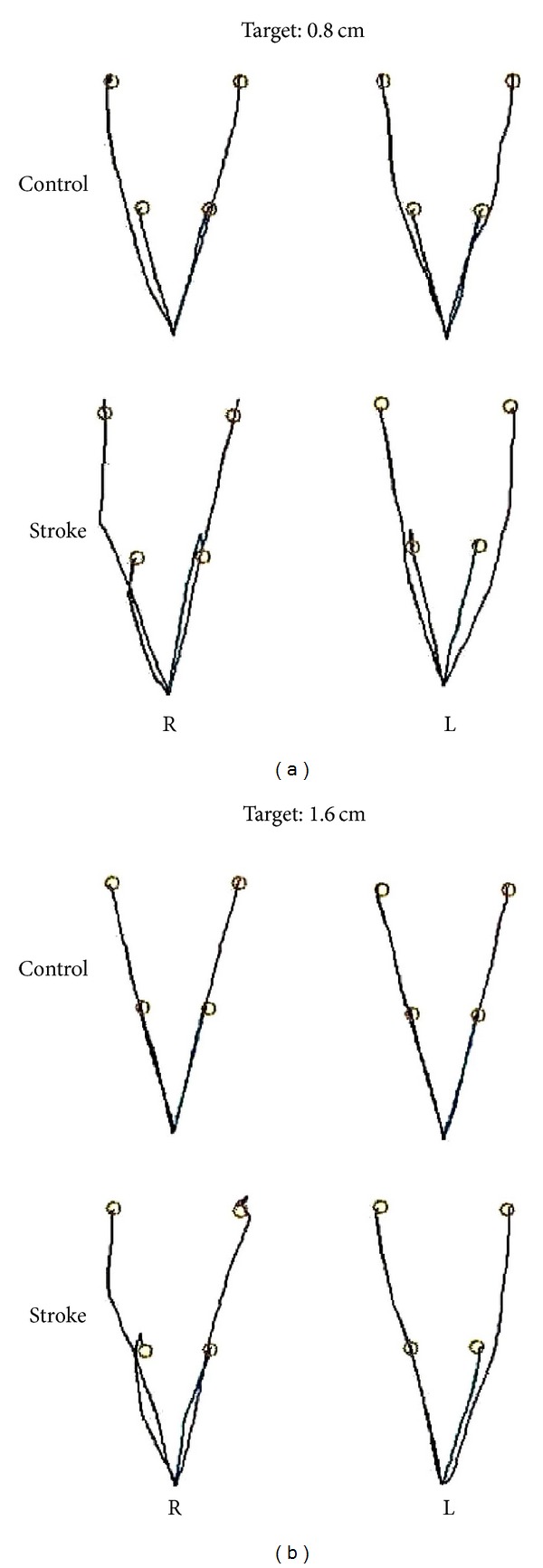
Trajectory profile from a representative participant of each group with the right (R) and left (L) arms for the two targets (0.8 and 1.6 cm) and two distances from the initial position (9 and 18 cm) used in the experiment.

Representative individual trajectory profiles performed by a control and right and left hemisphere damage participants are shown in Figure 2 for each task condition.

**Figure 3:**
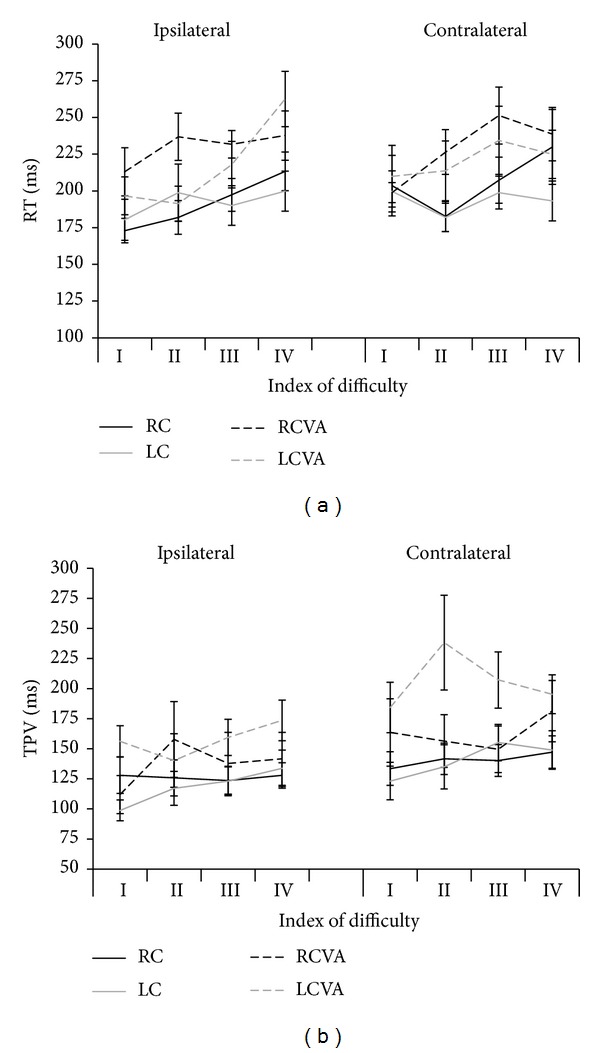
Mean reaction time (a) and mean time to peak of velocity (b) for the right (RC) and left (LC) arms of control group and ipsilesional arms of participants with right (RCVA) and left (LCVA) cerebrovascular accident for ipsilateral and contralateral movements for each index of difficulty (I = 3.5; II = 4.5; III = 4.5; IV = 5.5). Bars indicate standard error.

Mauchly's test was nonsignificant for all comparisons showing that the sphericity hypothesis was not violated. Mean values for reaction time (RT) in each ID for both ipsilateral and contralateral movements are shown in Figure 3(a). There was no main effect for group (RC: 199 ± 12 ms; LC: 193 ± 12 ms; RCVA: 229 ± 12 ms; LCVA: 219 ± 12 ms) or direction (ipsilateral: 208 ± 6 ms; contralateral: 212 ± 7 ms) for any comparison. A significant main effect for ID was obtained for all comparisons between groups (RC versus RCVA: *F*_2.6,46.3_ = 10.56; *P* < 0.0001; LC versus LCVA: *F*_2.5,44.9_ = 4.80; *P* = 0.008; RCVA versus LCVA: *F*_2.6,46.9_ = 10.18; *P* < 0.0001), such that the RT was greater for the highest ID (ID 3.5: 197 ± 7 ms; ID 4.5–9 cm: 202 ± 7 ms; ID 4.5–18 cm: 216 ± 7 ms; ID 5.5: 225 ± 8 ms). There was also a significant interaction between group and ID only for the comparison between RC and RCVA (*F*_2.57,46.3_ = 3.21; *P* = 0.038). The RT for the ID 4.5 (for both used distances) was higher for the right hemisphere damaged participants (9 cm: 232 ± 12 ms; 18 cm: 242 ± 12 ms) than for the control participants (9 cm: 182 ± 13 ms; 18 cm: 202 ± 12 ms).

The time to peak velocity (TPV) was significantly longer (*F*_1,18_ = 7.23; *P* = 0.02) for the LCVA (182 ± 14 ms) than for the LC (129 ± 14 ms), but it was similar for the other comparisons between groups (RC: 133 ± 16 ms versus RCVA: 150 ± 16 ms (*F*_1,18_ = 0.52; *P* = 0.48); LCVA versus RCVA (*F*_1,18_ = 1.54; *P* = 0.23)). It was also higher for the contralateral target (163 ± 6 ms) than for the ipsilateral (135 ± 5 ms) for all comparisons (RC versus RCVA: *F*_1,18_ = 5.14; *P* = 0.04; LC versus LCVA: *F*_1,18_ = 29; *P* < 0.0001; RCVA versus LCVA: *F*_1,18_ = 16.43; *P* = 0.001). TPV was similar for different ID (ID 3.5: 137 ± 8 ms; ID 4.5–9 cm: 152 ± 10 ms; ID 4.5–18 cm: 149 ± 8 ms; ID 5.5: 156 ± 7 ms), and no significant interactions were found across the analyzed factors (Figure 3(b)).

**Figure 4:**
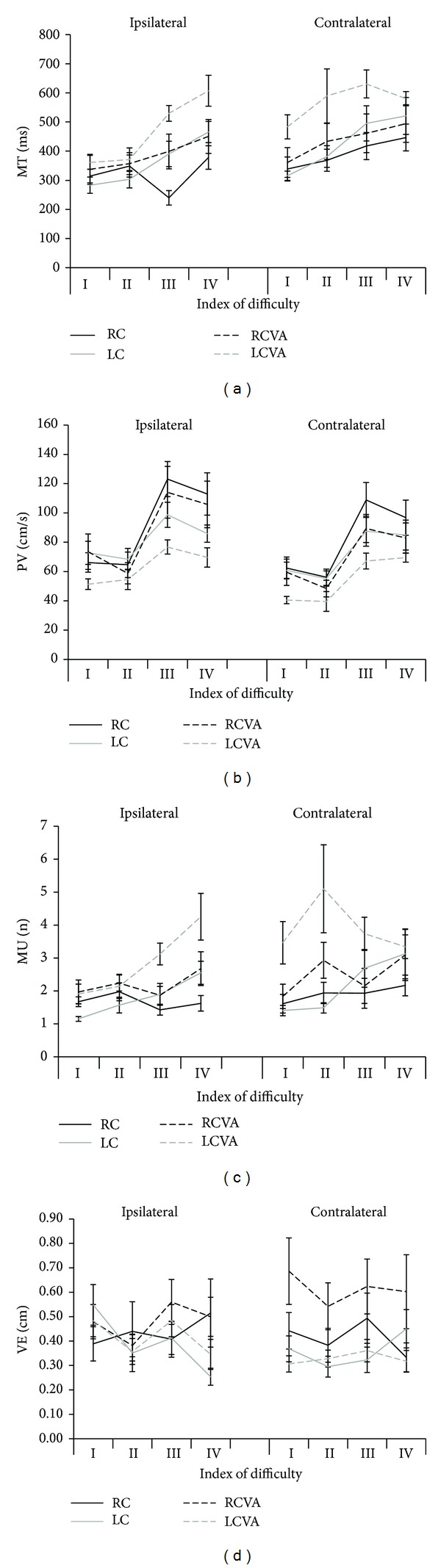
Mean values of movement time (a), peak of velocity (b), movement units (c), and variable error (d) for the right (RC) and left (LC) arms of control group and ipsilesional arms of participants with right (RCVA) and left (LCVA) cerebrovascular accident for ipsilateral and contralateral movements for each index of difficulty (I = 3.5; II = 4.5; III = 4.5; IV = 5.5). Bars indicate standard error.

The movement time (MT) was significantly higher (*F*_1,18_ = 6.95; *P* = 0.017) for the LCVA group (519 ± 33 ms) than for the LC group (395 ± 33 ms). The comparisons between the RC (357 ± 38 ms) and RCVA (412 ± 38 ms) groups (*F*_1,18_ = 0.88; *P* = 0.36) and between the left and right hemisphere damaged groups (*F*_1,18_ = 3.05; *P* = 0.1) did not show any difference. For all comparisons, the MT was significantly higher for the contralateral (457 ± 22 ms) target than the ipsilateral (384 ± 18 ms) target, but there was no interaction between group and direction. MT also increased with the ID (ID 3.5: 349 ± 17 ms; ID 4.5–9 cm: 394 ± 22 ms; ID 4.5–18 cm: 445 ± 22 ms; ID 5.5: 493 ± 22 ms). Even for the same ID 4.5, the MT was higher in the condition with larger target and longer distance than in the condition with smaller target and shorter distance, as can be seen in Figure 4(a). The relationship between TPV and MT, on the other hand, did not show any difference between groups (RC: 40 ± 2 ms; LC: 34 ± 2 ms; RCVA: 37 ± 2 ms; LCVA: 37 ± 2 ms) and directions (ipsilateral: 37 ± 1 ms; contralateral: 36.7 ± 1 ms), but there was a main effect for ID for the comparisons of RC versus RCVA (*F*_2.6,46_ = 4.01; *P* < 0.017), LC versus LCVA (*F*_2.5,45.5_ = 11.33; *P* < 0.0001), and RCVA versus LCVA (*F*_2.5,45.2_ = 9.39; *P* < 0.0001), indicating that the percentage of TPV/MT decreased with the increase of the ID, which means longer deceleration time for higher ID. There was a significant interaction between group and ID (*F*_2.6,46_ = 4.28; *P* = 0.013) for the RC versus RCVA comparison. The results showed that for the ipsilateral movement toward the larger target and longer distance, the RC group took more time to reach the PV than the RCVA group; that is, the RCVA group needed longer deceleration time for higher ID.

Similar to MT, the peak velocity (PV, *F*_1,18_ = 6.77; *P* = 0.018) and the smoothness (*F*_1,18_ = 7.89; *P* = 0.012) were significantly different between groups only for the comparison between the LC and LCVA groups (Figures 4(b) and 4(c)). The LCVA group was slower (59 ± 5 cm/s) and performed less smooth movements (3.4 ± 0.4 movement units, MU) than the LC group (77 ± 5 cm/s; 2 ± 0.4 MU). The other comparisons between groups were similar. The PV was higher and the movement was smoother for the ipsilateral than the contralateral movement for all groups. In relation to the ID, there was a main effect for all comparisons (RC versus RCVA: *F*_2.2,40_ = 52.8; *P* < 0.0001; LC versus LCVA: *F*_1.9,33.4_ = 30.52; *P* < 0.0001; RCVA versus LCVA: *F*_1.5,26.1_ = 50.55; *P* < 0.0001). The PV was higher for the ID with longer distances (18 cm) than for the ID with short distances (9 cm), independent of the target size. The smoothness was also influenced by the ID for all comparisons between groups (RC versus RCVA: *F*_1.5,27.6_ = 18.2; *P* < 0.018; LC versus LCVA: *F*_2.2,40.2_ = 9.21; *P* = 0.001; RCVA versus LCVA: *F*_1.8,33.1_ = 10.74; *P* = 0.010). There were more MU for the tasks with higher ID.

For the resultant variable error, there was a group difference only in the comparison of the RCVA versus LCVA (*F*_1,18_ = 5.40; *P* = 0.03). There was also a significant interaction between group and direction (*F*_1,18_ = 6.38; *P* = 0.021) for the RCVA versus LCVA comparison. The *post hoc* analysis showed that for the contralateral movement, the variable error was higher for the RCVA (0.61 ± 0.07 cm) than for the LCVA (0.33 ± 0.07 cm) group (Figure 4(d)).

## 4. Discussion

The purpose of this study was to verify the influence of the ID and direction on planning and execution of aiming movements and to analyze whether the side of the brain damage differentially affects the behavior of chronic stroke individuals. The results revealed that both planning and execution of ipsilesional movements are compromised by unilateral brain damage and that the behavior is altered, depending on the side of the lesion and on the task constraints.

The manipulation of the ID and the direction of the movement used in this study brought a visuospatial constraint for the task which affected both movement planning and execution for individuals with right brain damage. Although there were no differences between groups in the RT for all comparisons, the results showed a significant interaction between group and ID only for the comparison between RCVA and RC groups. RCVA participants took longer to start the movement in conditions where the combination of both dimensions and distances of the targets was used (i.e., ID = 4.5). It is interesting to note that in order to achieve the same ID, two different target dimensions were placed at two different distances from the initial position, which means that, for planning purposes, the visuospatial constraints of the task essentially affected the RCVA group.

It was found that the deceleration time increased with the increase of the ID for all groups, but the RC group took more time to reach the PV for the ipsilateral movement to the larger target placed on the longer distance. The RCVA group did not follow this behavior and showed larger deceleration time. Additionally, the RCVA group showed higher variable error than the LCVA group for all analyzed conditions, mainly for the contralateral movements. But, even taking longer to decelerate the movement, the RCVA group presented larger errors than the other groups. No differences between the RCVA and RC groups were found to be related to the MT, PV, or smoothness. The current results suggest that the visuospatial characteristics of the investigated experimental conditions caused an increased delay for planning the movement on right hemisphere damaged individuals, even keeping the movement less accurate.

Although the literature shows controversial findings on using the reaction time paradigm to assess laterality in motor planning on both healthy individuals [27–29] and those with unilateral brain damage [2, 30, 31], our results suggest that the performance degradation seen in individuals after a right hemispheric lesion can be attributed to the increased time necessary for the codification of spatial constraints of the task by this group. These results corroborate those found by Schaefer et al. [2], who showed longer RT for right hemisphere damaged group in aiming movements that varied with target directions than for left and right healthy control groups and left hemisphere damaged groups. These authors also found larger errors for the right lesion group, while the group with left hemisphere damage was similar to controls.

On the other hand, the different ID and directions of movement used in this study lead to a higher TPV, MT, and lower PV and smoothness on the individuals with left brain damage than the control group. No differences between RT and variable error were found between the LCVA and LC groups. These results were also found by others [1, 5, 21, 32] and suggest that the visuospatial constraints of the tasks affected the control of movement trajectory of the individuals with left hemisphere damage. This control must consider the purpose of the task, predict the external forces that act over the movement during its execution, and compensate for them [33], and it seems to be compromised on lesions of the left hemisphere [32, 34]. The fact that LCVA (compared to LC) took similar amounts of time to reach the PV reinforces the notion that deceleration time is more closely related to the accuracy and spatial constraints of the task and, therefore, compromised only after right brain damage. Because of the slower movement times found for all groups, which lasted more than 200 ms, it is possible that visually guided corrective movements occurred during the deceleration time. Even taking longer to decelerate than the control group, the RCVA group showed larger errors and only the trajectories of the LCVA group were less smooth. Besides that, most movements exhibited a single velocity peak. These results reinforce the notion about the dichotomy between spatial planning and modulation by the right hemisphere and execution by the left hemisphere.

The ID of the task influenced the RT, MT, and PV for all groups. These results support the notion that planning demand increases with the complexity of the task [11, 35, 36], which was determined in the present study by the combination of the distance and size of the target. Additionally, the movements performed to contralateral targets were longer, slower, and less smooth than the movements to ipsilateral targets, indicating that contralateral movements require higher interjoint coordination because of the higher movement amplitudes of the shoulder and elbow joints than those used in the ipsilateral movements [19, 37], which compromised the movement execution. The interesting point, however, was that the manipulation of the movement amplitude for the same ID brought differences on MT and PV only for the left hemisphere damage individuals. For the longer distance, these individuals took more time and were slower than the control group in completing the movement. Mutha and Sainburg [38], analyzing the variation of the acceleration amplitude and PV with the movement distance in healthy adults in targeted movements, showed a scaling effect of finger velocity with the movement distance. Our results suggest that this modulation according to the movement distance is affected for individuals with left hemisphere lesions.

In summary, the present findings suggest that the ID of the task and the direction of movement brought visuospatial constraints to the aiming tasks, which differentially affected the performance of individuals with left and right hemisphere damage following a stroke. The planning modulation according to the task constraints (reflected by longer latency for the movement onset and greater final position errors) is compromised by right hemisphere damage, while the trajectory execution (reflected by longer, slower, and less smooth movements) is more affected by left hemisphere damage. In addition, movement amplitude has a greater impact on aiming performance than the ID value itself, and the modulation of this performance according to the target distance is affected by left hemisphere damage. These results show that aspects like the direction and ID of the task on aiming movements should be considered for differential assessment and intervention in rehabilitation after right and left unilateral brain damage and point to the possibility of the usage of the digitizing tablet for more pragmatic and clinical studies.

## Figures and Tables

**Table 1:** Demographic and clinical data of the left and right poststroke hemiparesis and control groups.

	Control (*n* = 10)		RCVA (*n* = 10)	LCVA (*n* = 10)
Gender				
F/M	4/6		4/6	4/6
Age (years)				
Average	55		60	56
SD	8		7	9
Time after lesion (months)				
Average			52	47
SD			44	62
Fugl-Meyer Scale (score)				
Average			46.9	48.3
SD			14.1	19.1
MMSE (score)				
Average	28.5		27.8	26.1
SD	1.6		2.4	4.2

	Control (*n* = 10)		RCVA (*n* = 10)	LCVA (*n* = 10)
	Right	Left		

Pinch strength (kgf)				
Average	7.7	7.2	5.5	6.2
SD	1.5	1.1	1.6	1.6
Grip strength (kgf)				
Average	24.6	23.5	20.8	18.3
SD	3.3	4.7	4.3	4.2

RCVA: right cerebrovascular accident; LCVA: left cerebrovascular accident; MMSE: Mini-Mental State Exam; F: female; M: male; SD: standard deviation.
